# Comparison and phylogenetic analysis of the mitochondrial genomes of *Synodontis eupterus* and *Synodontis polli*

**DOI:** 10.1038/s41598-024-65809-4

**Published:** 2024-07-04

**Authors:** Cheng-He Sun, Chang-Hu Lu, Zi-Jian Wang

**Affiliations:** 1 The Co-Innovation Center for Sustainable Forestry in Southern China, College of Life Sciences, Nanjing Forestry University, Nanjing, 210037 China; 2 Agriculture and Rural Bureau of Gaochun District, Nanjing, 211300 China

**Keywords:** Mochokidae, Synodontis, Mitochondrial genome, Phylogenetic, Genetics, Genomics

## Abstract

We aimed to distinguish *Synodontis eupterus* and *Synodontis polli*. We performed sequencing and bioinformatic analysis of their mitochondrial genomes and constructed a phylogenetic tree of Mochokidae fish using maximum likelihood and Bayesian methods based on protein-coding gene (PCG) sequences of 14 Mochokidae species. The total length of the *S. eupterus* mitochondrial genome was 16,579 bp, including 13 (PCGs), 22 tRNA genes, two rRNA genes, and one D-loop, with an AT-biased nucleotide composition (56.0%). The total length of the *S. polli* mitochondrial genome was 16,544 bp, including 13 PCGs, 22 tRNA genes, two rRNA genes, and one D-loop, with an AT-biased nucleotide composition (55.0%). In both species, except for *COI*, PCGs use ATG as the starting codon, the vast majority use TAG or TAA as the ending codon, and a few use incomplete codons (T - or TA -) as the ending codon. Phylogenetic analysis showed that *S. eupterus* and *Synodontis clarias* converged into one branch, *S. polli* and *Synodontis petricola* converged into one branch, *Mochokiella paynei*, *Mochokus brevis*, and nine species of the genus *Synodontis* converged into one branch, and *M. paynei* clustered with the genus *Synodontis*. This study lays a foundation for rebuilding a clearer Mochokidae fish classification system.

## Introduction

*Synodontis* (Cuvier, 1816) (Teleostei, Mochokidae) is a popular fish in aquariums. It is important to correctly identify these fish, especially in fishing competitions where species identification can affect records. However, due to the unreliable taxonomic nature of prominent morphological features, certain species are extremely difficult to identify^1^. The genus *Synodontis* belongs to the family Mochokidae, which is abundant in all the three East African Great Lakes. Therefore, it is an ideal system for comparing data from phylogenetic and geographic analyses with existing data on the family Cichlidae. Among the nine non-cichlid families shared by the three East African Great Lakes, Mochokidae shows the closest overlap in habitat with cichlids, as it mainly occurs in coastal and sub-coastal regions^2^. In East African mochokids, *Synodontis* is more diverse in species than *Chiloglanis*. Moreover, most *Synodontis* species are omnivorous, which enables them to cope with seasonal changes in food richness and habitat^3,4^. Compared to many more specialized fish species, such as cichlids, which adapt to lakes, their feeding habits broaden their food niche and enable them to better settle in different habitats.

In recent years, DNA barcode technology has gradually developed as a molecular identification method^5^. In the process of species identification in animals and plants, DNA barcode technology mainly uses a *COI* gene sequence that is approximately 650 bp long to distinguish species, gradually realizing automation and standardization of the identification process, thereby reducing the dependence of species identification on traditional, empirical morphological classification methods. *COI*, an important mitochondrial gene, is characterized by maternal inheritance, simple structure, moderate evolutionary rate, high polymorphism, and easy amplification using universal primers^6^. It is commonly used for species identification and analysis of phylogenetic relationships between closely related species, subspecies, and geographic populations. Currently, DNA barcode technology is widely used for species identification of freshwater fish^7^ and marine fish^8^, with a recognition rate of over 90%^9^. In addition, DNA barcode technology has shown significant advantages in the identification of species and evaluation of their genetic diversity^10^.

The mitochondrial genome of animals has typical genetic characteristics, such as maternal inheritance patterns, conservative coding regions, fast evolution of control regions, high mutation rates, and independent replication units, making it a very useful material for molecular evolutionary research. The mitochondrial genome of the vast majority of metazoans is a double-stranded closed circular DNA molecule, with a size of 14–20 kb, encoding a total of 37 genes, including 22 transfer RNA (tRNA) genes, 13 protein-coding genes (PCGs), and two ribosomal RNA (rRNA) genes^11^. Additionally, there is a long non-coding region in the mitochondrial genome called the control region (D-loop)^12^.

Currently, mitochondrial genome data are widely used to study phylogenetic relationships at different taxonomic levels in bony fish^13^. However, there are few comparative studies based on mitochondrial genome data from Mochokidae. Therefore, this study focused on *Synodontis eupterus* and *Synodontis polli* and measured their mitochondrial genome sequences. The structural characteristics of the mitochondrial genome sequences were also analyzed, and using the mitochondrial genome sequences of 12 Mochokidae species published in the GenBank database, a phylogenetic tree was constructed to provide a basis for further research on their genetic evolution and classification.

## Materials and methods

### Sample collection and DNA extraction

All methods were performed in accordance with the relevant guidelines. All specimens in this study were collected in accordance with Chinese laws. The collection and sampling of the specimens were reviewed and approved by the Animal Ethics Committee of Nanjing Forestry University. All experiments were conducted with respect to animal welfare and care. The study complied with CBD and Nagoya protocols and with the ARRIVE guidelines (https://arriveguidelines.org). Both fish samples were sourced from flower and bird markets in Fuzimiao, Qiqiao Weng, Nanjing, Jiangsu Province, China. After morphological identification, muscle tissue samples from both were taken and stored at − 80 °C for genomic DNA extraction. Approximately 50 mg of *S. eupterus* and *S. polli* muscle tissues was taken and used for genomic DNA extraction using a TIANamp Genomic DNA Kit blood/cell/tissue genomic DNA extraction kit (DP201101X, TIANGEN). The purity and concentration of the DNA were determined using an ultraviolet spectrophotometer, while the integrity was determined by 1% agarose gel electrophoresis. To ensure accuracy in sample identification, universal primers were used to amplify the Cytb, COI, and 16S rRNA genes.

### Genomic sequencing

DNA was fragmented by mechanical interruption (ultrasonic), purified, and end-repaired, followed by the addition of A at the 3′ end and connection to a sequencing connector. Agarose gel electrophoresis was used to select the size of the fragments, followed by PCR and enrichment analysis, and a sequencing library was constructed. The constructed library was purified to remove connector pollution, and a library quality inspection was carried out. The qualified libraries were sequenced using the Illumina NovaSeq platform.

### Sequence assembly and analysis

To reduce the complexity of sequence assembly, bowtie2 v2.2.4 software^14^, which is a very sensitive local software, was used for alignment with the mitochondrial genome database, and the aligned sequence was used as the mitochondrial genome sequence (mtDNA sequence). The mitochondrial genome was assembled using the SPAdes^15^ software, without relying on the reference genome. Quality control was performed after the assembly was completed using the reference sequence of *Synodontis petricola* MZ930090 (https://www.ncbi.nlm.nih.gov/nuccore/MZ930090). Using Mitos2^16^ (http://mitos2.bioinf.uni-leipzig.de), we annotated the assembled sequences, compared the Mitos2 annotation results with those of related species, corrected the standards, and obtained the final annotation results. A mitochondrial genome map was created using MitoFish (https://mitofish.aori.u-tokyo.ac.jp/). Comparative analysis of mitochondrial genome structure for close-source species was performed using the PhyloSuite v1.2.1 software^17^.

### Systematic evolution analysis

The complete mitochondrial genome sequences of Mochokidae were downloaded from NCBI, with *Corydoras aeneus* MZ571336 and *Austroglanis sclateri* MZ930070 as outgroups. We performed evolutionary tree analysis using tandem sequences of 13 PCGs, performed multiple sequence alignment using MAFFT v7.313 software, optimized the results through MACSE v2.03 comparison, and then performed tandem analysis after block pruning. Using ModelFinder for partition model prediction, maximum likelihood phylogenies were inferred using IQ-TREE^18^, and Bayesian inference phylogenies were inferred using MrBayes 3.2.6^19^.

## Results

### Basic characteristics of mitochondrial genome

**Figure 1:**
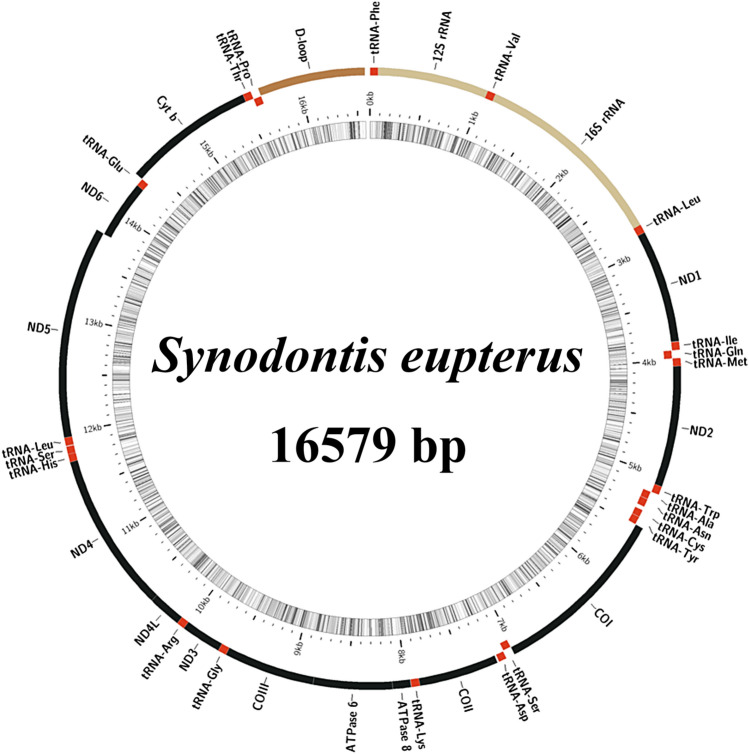
Mitochondrial genome map of *Synodontis eupterus*.

**Figure 2:**
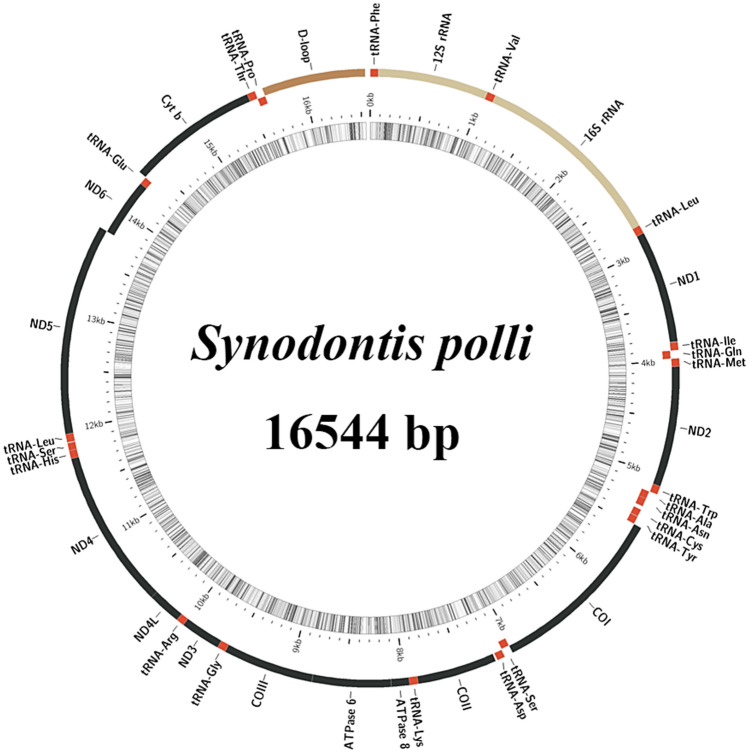
Mitochondrial genome map of *Synodontis polli*.

The mitochondrial genome sizes of *S. eupterus* and *S. polli* were 16,579 and 16,544 bp (Supplementary Information 1 and 2), respectively, with a total of 37 genes, including 13 PCGs, 22 tRNA genes, and 2 rRNA genes (Figs. 1 and 2). Analysis of the nucleotide composition of *S. eupterus* and *S. polli* showed that the A + T content of *S. eupterus* accounted for 56.0% of the entire mitochondrial genome, whereas the A + T contents of PCGs, tRNA, and rRNA accounted for 55.8, 56.5, and 54.3% of the entire mitochondrial genome, respectively (Table 1). *S. polli* A + T content accounted for 55.0% of the entire mitochondrial genome, whereas the A + T content of PCGs, tRNAs, and rRNAs accounted for 54.6, 56.7, and 53.9% of the entire mitochondrial genome, respectively (Table 2).

**Table 1:** Nucleotide composition of *Synodontis eupterus* mitochondrial genome.

*S.eupterus*	Size (bp)	T/%	C/%	A/%	G/%	AT (%)	GC (%)	AT skewness	GC skewness
Full genome	16,579	24.6	28.8	31.4	15.2	56.0	44.0	0.121	− 0.309
PCGs	11,397	26.1	29.4	29.7	14.8	55.8	44.2	0.065	− 0.330
tRNAs	1561	27.7	20.6	28.8	22.9	56.5	43.5	0.019	0.053
rRNAs	2643	20.4	25.7	33.9	20.0	54.3	45.7	0.249	− 0.125
CR	931	31.6	22.4	29.3	16.6	60.9	39.0	− 0.038	− 0.149

**Table 2:** Nucleotide composition of *Synodontis polli* mitochondrial genome.

*S.polli*	Size (bp)	T(U)/%	C/%	A/%	G/%	AT (%)	GC (%)	AT skewness	GC skewness
Full genome	16,544	24.5	29.1	30.5	15.8	55.0	44.9	0.109	− 0.296
PCGs	11,397	26.1	29.7	28.5	15.6	54.6	45.3	0.044	− 0.311
tRNAs	1562	27.8	20.3	28.9	23.0	56.7	43.3	0.019	0.062
rRNAs	2640	20.3	25.8	33.6	20.2	53.9	46.0	0.247	− 0.122
CR	898	31.3	22.8	29.6	16.1	60.9	38.9	− 0.028	− 0.172

Previous studies have shown that base composition skewness plays an important role in transcription and replication. The AT skewness of the *S. eupterus* mitochondrial genome (0.121) was similar to that of the *S. polli* (0.109) mitochondrial genome, indicating that the content of adenine (As) was higher than that of thymine (Ts). The negative skewness (− 0.309) of the mitochondrial genome GC of *S. eupterus* was lower than that of *S. polli* GC (− 0.296), indicating that the cytosine (Cs) content was higher than that of guanine (Gs) (Tables 1 and 2).

Two overlapping gene regions, with sizes of 7 bp and 10 bp, were found in the mitochondrial genome of *S. eupterus*. Overlapping fragments in genome are generally only 7–10 bp in fish, whereas in mammals they can generally reach 40–46 bp^20^. The mitochondrial genome of *S. eupterus* has 11 gene intervals, with a length distribution of 1–32 bp. The interval between tRNA-Asn and tRNA-Cys was the longest at 32 bp (Table 3).

**Table 3:** Mitochondrial genome characteristics of *Synodontis eupterus*.

Gene	Strand	Position		Size	Intergenic nucleotides	Codon	
		From	To			Start	Stop
tRNA-Phe	+	1	70	70	0		
12S rRNA	+	71	1028	958	0		
tRNA-Val	+	1029	1100	72	0		
16S rRNA	+	1101	2785	1685	0		
tRNA-Leu2	+	2786	2860	75	0		
ND1	+	2861	3835	975	0	ATG	TAA
tRNA-Ile	+	3838	3909	72	2		
tRNA-Gln	−	3909	3979	71	− 1		
tRNA-Met	+	3979	4048	70	− 1		
ND2	+	4049	5093	1045	0	ATG	T
tRNA-Trp	+	5094	5166	73	0		
tRNA-Ala	−	5169	5237	69	2		
tRNA-Asn	−	5239	5311	73	1		
tRNA-Cys	−	5344	5409	66	32		
tRNA-Tyr	−	5412	5481	70	2		
COI	+	5483	7033	1551	1	GTG	TAA
tRNA-Ser2	−	7034	7104	71	0		
tRNA-Asp	+	7109	7178	70	4		
COII	+	7193	7883	691	14	ATG	T
tRNA-Lys	+	7884	7957	74	0		
ATP8	+	7959	8126	168	1	ATG	TAA
ATP6	+	8117	8799	683	− 10	ATG	TA
COIII	+	8800	9583	784	0	ATG	T
tRNA-Gly	+	9584	9657	74	0		
ND3	+	9658	10,006	349	0	ATG	T
tRNA-Arg	+	10,007	10,076	70	0		
ND4L	+	10,077	10,373	297	0	ATG	TAA
ND4	+	10,367	11,747	1381	− 7	ATG	T
tRNA-His	+	11,748	11,817	70	0		
tRNA-Ser1	+	11,818	11,884	67	0		
tRNA-Leu1	+	11,889	11,961	73	4		
ND5	+	11,962	13,788	1827	0	ATG	TAA
ND6	−	13,785	14,300	516	− 4	ATG	TAG
tRNA-Glu	−	14,301	14,369	69	0		
Cytb	+	14,371	15,508	1138	1	ATG	T
tRNA-Thr	+	15,509	15,580	72	0		
tRNA-Pro	−	15,579	15,648	70	− 2		
D-loop	+	15,649	16,579	931	0		

In the mitochondrial genome of *S. polli*, two gene regions overlapped with neighboring genes, with overlapping region lengths of 7 and 10 bp. The mitochondrial genome of *S. polli* consists of 11 gene-spacer regions, with nucleotide lengths ranging from 1 to 32 bp. The interval between tRNA-Asn and tRNA-Cys was the longest at 32 bp (Table 4).

**Table 4:** Mitochondrial genome characteristics of *Synodontis polli*.

Gene	Strand	Position		Size	Intergenic nucleotides	Codon	
		From	To			Start	Stop
tRNA-Phe	+	1	70	70	0		
12S rRNA	+	71	1028	958	0		
tRNA-Val	+	1029	1100	72	0		
16S rRNA	+	1101	2782	1682	0		
tRNA-Leu2	+	2783	2857	75	0		
ND1	+	2858	3832	975	0	ATG	TAG
tRNA-Ile	+	3835	3906	72	2		
tRNA-Gln	−	3906	3976	71	− 1		
tRNA-Met	+	3976	4045	70	− 1		
ND2	+	4046	5090	1045	0	ATG	T
tRNA-Trp	+	5091	5163	73	0		
tRNA-Ala	−	5166	5234	69	2		
tRNA-Asn	−	5236	5308	73	1		
tRNA-Cys	−	5341	5406	66	32		
tRNA-Tyr	−	5409	5478	70	2		
COI	+	5480	7030	1551	1	GTG	TAA
tRNA-Ser2	−	7031	7101	71	0		
tRNA-Asp	+	7106	7175	70	4		
COII	+	7190	7880	691	14	ATG	T
tRNA-Lys	+	7881	7954	74	0		
ATP8	+	7956	8123	168	1	ATG	TAA
ATP6	+	8114	8796	683	− 10	ATG	TA
COIII	+	8797	9580	784	0	ATG	T
tRNA-Gly	+	9581	9654	74	0		
ND3	+	9655	10,003	349	0	ATG	T
tRNA-Arg	+	10,004	10,073	70	0		
ND4L	+	10,074	10,370	297	0	ATG	TAA
ND4	+	10,364	11,744	1381	− 7	ATG	T
tRNA-His	+	11,745	11,815	71	0		
tRNA-Ser1	+	11,816	11,882	67	0		
tRNA-Leu1	+	11,887	11,959	73	4		
ND5	+	11,960	13,786	1827	0	ATG	TAA
ND6	−	13,783	14,298	516	− 4	ATG	TAG
tRNA-Glu	−	14,299	14,367	69	0		
Cytb	+	14,369	15,506	1138	1	ATG	T
tRNA-Thr	+	15,507	15,578	72	0		
tRNA-Pro	−	15,577	15,646	70	− 2		
D-loop	+	15,647	16,544	898	0		

Although the genome was arranged compactly as a whole, 11 gene intervals were found in both the *S. eupterus* and *S. polli* mitochondrial genomes, with a total length of 64 bp each. The longest interval was 32 bp, and the shortest interval was 1 bp, which was found in multiple locations.

Thirteen PCGs with a total length of 11,397 bp were obtained from the entire mitochondrial genome of *S. eupterus*, with an A + T content of 55.8%. Among the 13 PCGs, 12 (*NDl*, *ND2, COI, COII, ATP6, ATP8, COIII, ND3, ND4L, ND4, ND5,* and *Cytb*) are located on the heavy chain, and one (*ND6*) is located on the light chain. Except for *COI*, which uses GTG as the starting codon, all the other PCGs use ATG as the starting codon. This is common in the mitochondrial genomes of other vertebrates^21^. *COI, ATP8, ND4L,* and *ND5* use TAA as the termination codon; *ND1* and *ND6* use TAG as the termination codon; *ATP6* is encoded by the incomplete termination codon TA-; and *COII, COIII, Cytb, ND2, ND3, and ND4* are encoded by the incomplete termination codon T– (Table 3).

The mitochondrial genome of *S. polli* contains 13 PCGs, with a length of 11,397 bp and an A + T content of 54.6%. Except for *COI*, which uses GTG as the starting codon, all other PCGs use ATG as the starting codon, which is consistent with the characteristics of *S. eupterus*. *COI, ATP8, ND1, ND4L, and ND5* use TAA as the termination codon; *ND6* uses TAG as the termination codon; *ATP6* is encoded by the incomplete termination codon TA-; and *COII, COIII, Cytb, ND2, ND3, and ND4* are encoded by the incomplete termination codon T (Table 4). The similarity between the genes of *S. eupterus* and *S. polli* is as high as 91%, indicating a close genetic relationship.

### tRNA and rRNA

tRNAs play a crucial role in the adaptation of molecules during protein synthesis. The total length of tRNAs in *S. eupterus* was 1561 bp, with an A + T content of 56.5%, AT skewness of 0.019, and GC skewness of 0.053. The rRNA length of this species is 2643 bp, with an A + T content of 54.3%. The percentage of As (33.9%) was higher than that of Ts (20.4%), resulting in an AT skewness of 0.249. Similarly, the percentage of Gs (20.0%) was lower than that of Cs (25.7%), resulting in a negative GC bias of 0.125 (Table 1). It was predicted that there were 22 tRNAs in S. *eupterus*, with a length range of 66–75 bp.

The tRNA length of *S. polli* was 1562 bp, with an A + T content of 56.7%, AT bias of 0.019, and GC bias of 0.062. The rRNA length of *S. polli* is 2640 bp, with an A + T content of 53.9%. The percentage of As (33.6%) in *S. polli* is higher than that of Ts (20.3%), resulting in an AT bias of 0.247, whereas the percentage of Gs (20.2%) was lower than that of Cs (25.8%), resulting in a negative GC bias of 0.122 (Table 2). It was predicted that there are 22 tRNAs in the *S. polli* mitochondrial genome, with a length range of 66–75 bp. Post-transcriptional processing and modification of tRNA molecules can decrease their stability^22^, resulting in changes in their structure.

The interactions between codons and anticodons are significantly influenced by the modification position, which is usually near the swinging position. This characteristic is well-preserved in eukaryotes and directly affects the regulation of translation efficiency, transfer, and maintenance^23^. The stability of tRNAs depends on the changes in their central tRNA structure, which may lead to tRNA degradation and differentiation.

### Control region and codon usage *bias*

**Figure 3:**
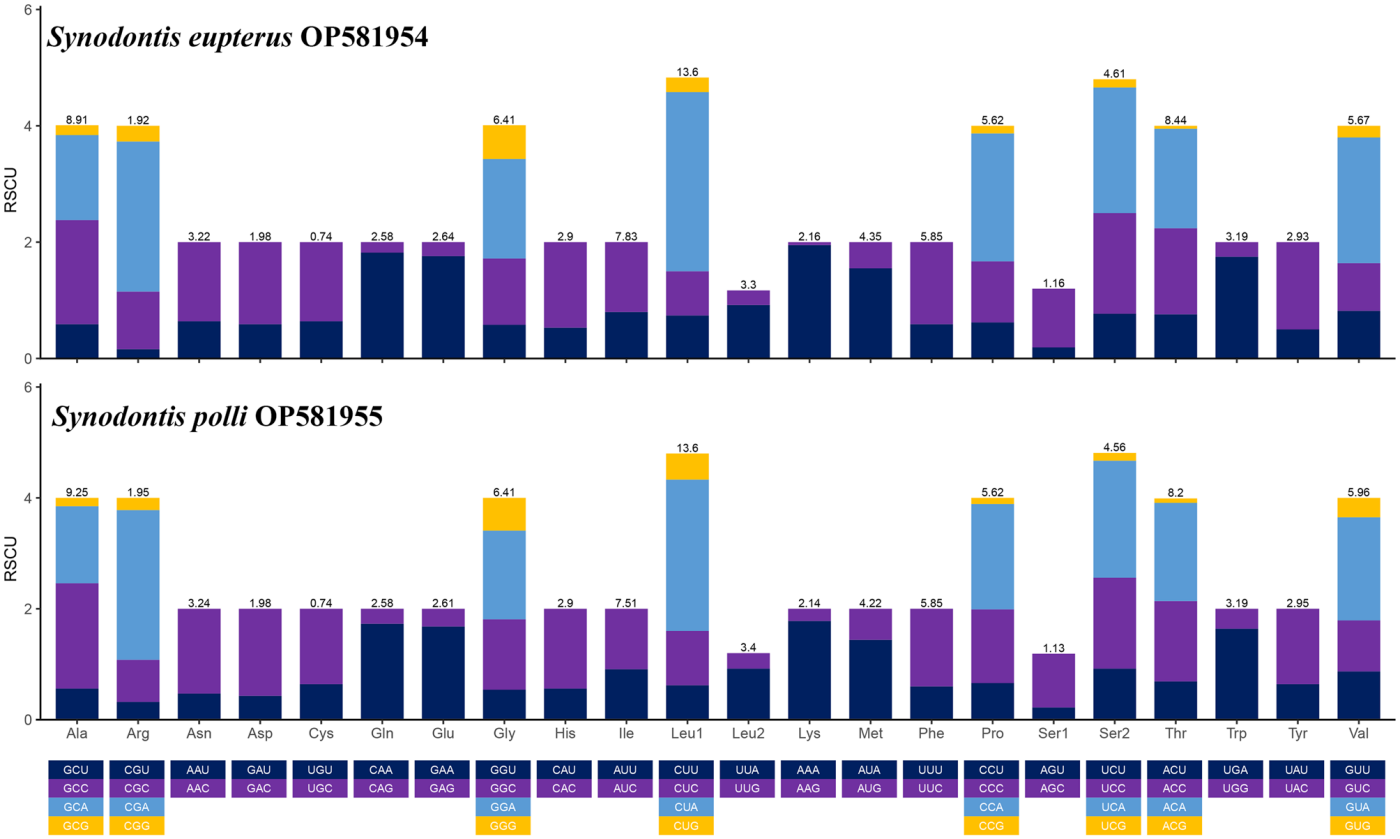
Codon usage of protein-coding genes of *Synodontis eupterus* and* Synodontis polli*.

Sequencing revealed that the mitochondrial genomes of *S. eupterus* and *S. polli* each contain control regions. The D-Loop has the fastest evolution rate in the mitochondrial genome, and its sequence variation is also the highest^24^. This study compared and analyzed the control region sequences of the two species and found that the control region lengths of *S. eupterus* and *S. polli* were 931 bp and 898 bp, respectively, with an A + T content of 60.9% for the control regions of both species. Previous studies have shown that mitochondrial DNA is important for studying various fish species, including bony fishes. They used control regions as markers for studying intra-species variation, which varies in many vertebrates, such as bony fish^25^, humans^26^, and birds^27^. The relative synonymous codon usage (RSCU) of amino acid utilization in the mitochondrial genomes of *S. eupterus* and *S. polli* is shown in Fig. 3.

The RSCU analysis showed that the mitochondrial genomes of *S. eupterus* and *S. polli* had the highest frequency of the amino acid codons for Leu, Ala, Thr, Ile, and Gly, with fewer Cys amino acid codons. The number of hydrophobic amino acid codons in the vertebrate mitochondrial genome is higher than the number of hydrophilic amino acid codons^28^. This indicates that the genomic regions close to the D-loop are extensively utilized and exhibit high translation efficiency, and can be effectively translated into the vertebrate mitochondrial genome.

### Phylogenetic analysis

**Figure 4:**
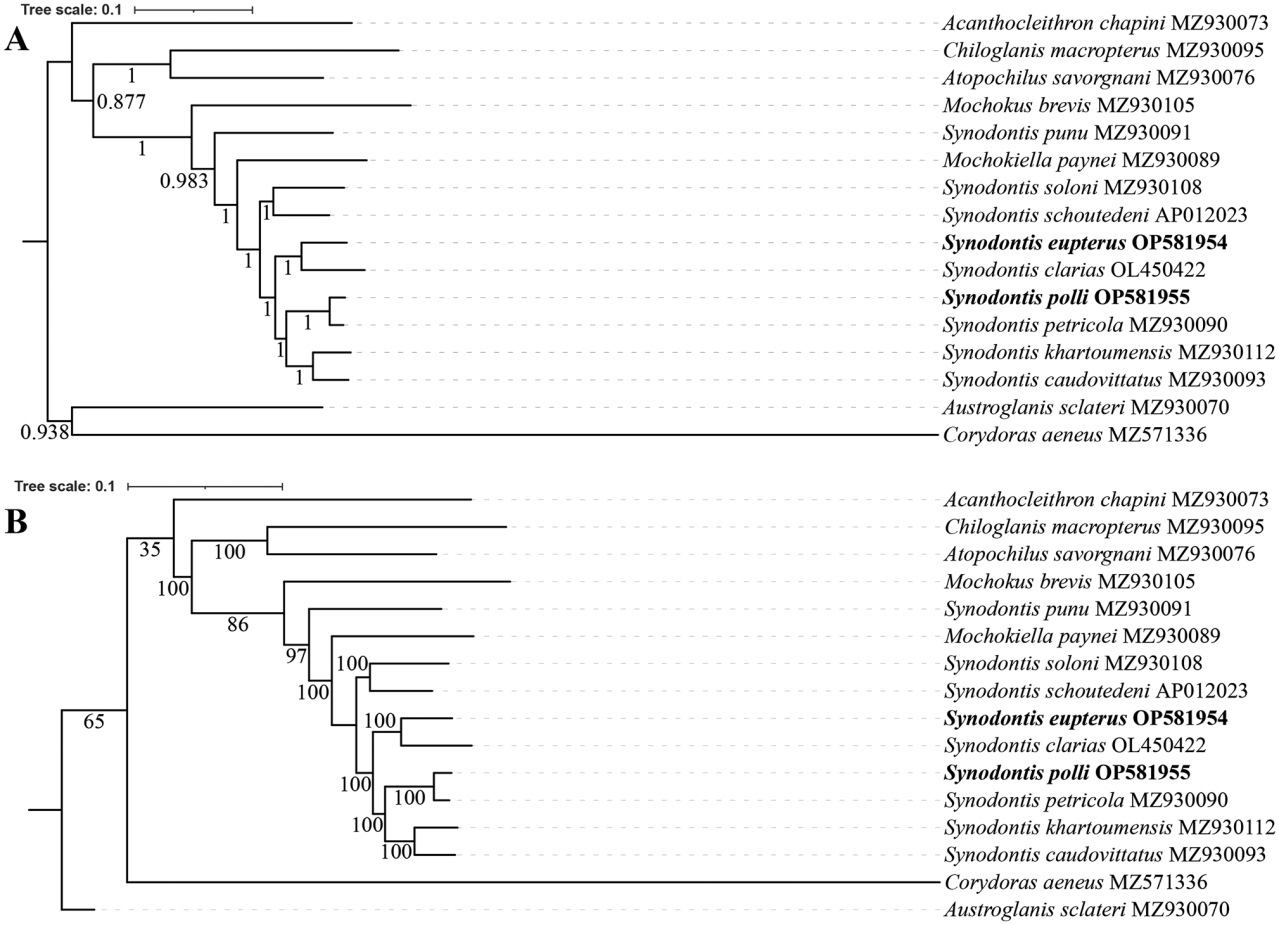
Bayesian inference (BI) (**A**) and maximum likelihood (ML) (**B**) phylogenetic trees based on the nucleotide datasets for 13 protein-coding genes from the mitogenomes of 14 Mochokidae fishes and two outgroups. The numbers along the branches indicate the Bayesian posterior probability values and ML bootstrap values, respectively.

We constructed phylogenetic trees of 14 Mochokidae fish species, including *S. eupterus* and *S. polli,* and two outgroup species based on PCG data. The phylogenetic trees constructed using the two methods had the same topological structure (Fig. 4). The results showed that the target species *S. eupterus* and *Synodontis clarias* clustered together, which confirmed the results of Dayet al.^29^. The other target species, *S. polli* and *S. petricola*, clustered into one branch, which is consistent with previous research results^30–32^. It is worth noting that *Mochokiella paynei*, *Mochokus brevis*, and 9 species of *Synodontis* genus converged into one branch, and *M. paynei* clustered into the genus *Synodontis*; a similar phenomenon was observed by Schedel et al.^33^.

## Discussion

This study systematically analyzed the structural characteristics, base composition, codon preferences, and PCGs of the mitochondrial genomes of *S. eupterus* and *S. polli*. The results indicated that *S. eupterus* and *S. polli* have a significant AT preference, which is similar to the base composition of vertebrate mitochondrial genomes^34^. The G-base content in *S. eupterus* and *S. polli* is similar to that in other bony fish, such as *Sillago aeolus* (18.75%)^35^ and *Oryzias celebensis* (17.60%)^36,37^, indicating significant anti-guanine effects^20^. In the mitochondrial genomes of *S. eupterus* and *S. polli*, all 13 PCGs, except *ND6*, were located on the heavy chain. In the codon preference analysis, the ATG codon usage frequency was the highest. The results of our analysis of start and end codons, lengths, and PCGs of the 37 genes in the mitochondrial genomes of *S. eupterus* and *S. polli* were consistent with the conclusions drawn by previous researchers^38^.

During mitochondrial whole-genome sequencing, mtDNA is prone to mutations and is difficult to repair, and the method for indirectly obtaining mtDNA information from high-throughput sequencing data has an important problem of sequence contamination^39^, which leads to erroneous research and inference of biodiversity, population genetics, species phylogenetic relationships, and mitochondrial diseases. To avoid contamination, we used various methods, such as single fragment extension and direct mapping of near source species, to assemble the mitochondrial genome. In this study, we sequenced the entire mitochondrial genomes of *S. eupterus* and *S. polli*, revealing the genetic characteristics and differentiation of the related species. By combining morphological^1–4^ and bioinformatic analyses, *S. eupterus* and *S. polli* were accurately distinguished.

mtDNA has a fast evolution rate and is a good source of genetic material. Phylogenetic studies of mtDNA have been widely used to outline relationships between species^40^. However, when studying small domains, there are limited data and information obtained from mtDNA; therefore, taxonomy is considered the foundation for understanding biodiversity and evolutionary behavior. Phylogenetic analysis was used to compare and study the similarities and differences within a family^41^. At present, phylogenetic trees constructed by the maximum likelihood and Bayesian methods are widely accepted, and this study combined these two methods to predict phylogeny. We found that *S. eupterus* and *Synodontis clarias* clustered together, while *S. polli* and *S. petricola*, clustered into one branch, consistent with previous studies^29–31^. Moreover, *Mochokiella paynei*, *Mochokus brevis*, and 9 species of *Synodontis* genus converged into one branch, and *M. paynei* clustered into the genus *Synodontis*; consistent with previous observations^33^.

Wong et al.^42^ used DNA barcode technology to detect three types of cod ingredients in 96 fish and seafood products extracted from markets and restaurants in northeastern North America, including *Gadus morhua*, *Theragra chalcogramma*, and *Merluccius paradoxus*. Lakra et al.^43^ analyzed the phylogenetic evolution of 115 fish species in the Indian Ocean using *COI* genes and found that the traditional taxonomic characteristics of the groups formed by *COI* genes in the NJ evolutionary tree were consistent, and the phylogenetic relationships between these groups were well revealed. Mat et al.^44^ sequenced a 36-bp long *COI* fragment from 723 individuals of 652 hypothesized species of the family Carangidae distributed in the waters of the Malay Archipelago in India and compared the variability of mitochondrial DNA *COI* fragments within and between species to evaluate the applicability of *COI* fragments for species identification. They found that all species formed monophyletic clusters in the phylogenetic tree, indicating that DNA barcode technology has high application value in fish species identification. This study was based on the mitochondrial genome, and it identified base differences in the *COI* genes of *S. eupterus* and *S. polli*. Specific primers will thus be designed based on this and the two will be identified using DNA barcode technology.

The evolution rate of protein coding genes is moderate, and different protein coding genes exhibit different evolutionary characteristics. They can be grouped based on the phylogenetic relationships of genes, but the results obtained from different groups are not the same. Therefore, a systematic analysis of each group is necessary. The DNA sequence of mitochondrial coding genes is the preferred gene for phylogenetic analysis, and the effective population size is one-quarter of that of nuclear autosomal genes. Therefore, gene trees constructed based on mitochondria have a higher probability of consistency with species trees than those constructed on nuclear autosomes. Therefore, they are often used to estimate the development history of recent evolutionary groups^45^. However, the functional differences of different genes may lead to different intensities of natural selection throughout history, resulting in the use of different genes in molecular phylogenetic analysis to obtain completely different gene trees. Therefore, compared to phylogenetic trees constructed with DNA barcodes or other single genes, phylogenetic trees constructed with mitochondrial whole genome exhibit more optimized stability and accuracy.

## Conclusion

This study involved phylogenetic analysis of mitochondrial genomes to accurately distinguish *S. eupterus* and *S. polli*, laying a foundation for the establishment of a clearer classification system for Mochokidae fish and providing new directions for further classification research. The mitochondrial genome sizes of *S. eupterus* and *S. polli* were 16,579 and 16,544 bp, respectively, with a total of 37 genes, including 13 PCGs, 22 tRNA genes, and 2 rRNA genes. Phylogenetic trees constructed using the maximum likelihood and Bayesian methods are widely accepted, and were combined in the present study to predict phylogeny. We found that *S. eupterus* and *Synodontis clarias* clustered together, while *S. polli* and *S. petricola*, clustered into a single branch.

## Supplementary Information

Supplementary Information 1.

Supplementary Information 2.

## Data Availability

The complete mitochondrial genome sequences and annotations of *Synodontis eupterus* and *Synodontis polli* are available in the National Center for Biotechnology Information (NCBI) GenBank database https://www.ncbi.nlm.nih.gov/genbank/), and accession numbers OP581954 and OP581955, respectively.
