# Author Correction: Comparison and phylogenetic analysis of the mitochondrial genomes of *Synodontis eupterus* and *Synodontis polli*

**DOI:** 10.1038/s41598-024-81891-0

**Published:** 2024-12-10

**Authors:** Cheng‑He Sun, Chang‑Hu Lu, Zi‑Jian Wang

**Affiliations:** 1 The Co-Innovation Center for Sustainable Forestry in Southern China, College of Life Sciences, Nanjing Forestry University, Nanjing, 210037 China; 2 Agriculture and Rural Bureau of Gaochun District, Nanjing, 211300 China

Correction to: *Scientific Reports* 10.1038/s41598-024-65809-4, published online 04 July 2024

In the original version of this Article, Cheng-He Sun was incorrectly listed as a corresponding author. The sole corresponding author for this Article is Chang-Hu Lu. Correspondence and request for materials should be addressed to luchanghu@njfu.com.cn

The original Article has been corrected.

